# An Improved BeiDou-2 Satellite-Induced Code Bias Estimation Method

**DOI:** 10.3390/s18051354

**Published:** 2018-04-27

**Authors:** Jingyang Fu, Guangyun Li, Li Wang

**Affiliations:** Zhengzhou Institute of Surveying and Mapping Zhengzhou 450000, China; guangyun_li@sohu.com (G.L.); 1111820221@hit.edu.cn (L.W.)

**Keywords:** BeiDou, code multipath bias, multipath combination, precise point positioning, wide-lane fractional cycle bias

## Abstract

Different from GPS, GLONASS, GALILEO and BeiDou-3, it is confirmed that the code multipath bias (CMB), which originate from the satellite end and can be over 1 m, are commonly found in the code observations of BeiDou-2 (BDS) IGSO and MEO satellites. In order to mitigate their adverse effects on absolute precise applications which use the code measurements, we propose in this paper an improved correction model to estimate the CMB. Different from the traditional model which considering the correction values are orbit-type dependent (estimating two sets of values for IGSO and MEO, respectively) and modeling the CMB as a piecewise linear function with a elevation node separation of 10°, we estimate the corrections for each BDS IGSO + MEO satellite on one hand, and a denser elevation node separation of 5° is used to model the CMB variations on the other hand. Currently, the institutions such as IGS-MGEX operate over 120 stations which providing the daily BDS observations. These large amounts of data provide adequate support to refine the CMB estimation satellite by satellite in our improved model. One month BDS observations from MGEX are used for assessing the performance of the improved CMB model by means of precise point positioning (PPP). Experimental results show that for the satellites on the same orbit type, obvious differences can be found in the CMB at the same node and frequency. Results show that the new correction model can improve the wide-lane (WL) ambiguity usage rate for WL fractional cycle bias estimation, shorten the WL and narrow-lane (NL) time to first fix (TTFF) in PPP ambiguity resolution (AR) as well as improve the PPP positioning accuracy. With our improved correction model, the usage of WL ambiguity is increased from 94.1% to 96.0%, the WL and NL TTFF of PPP AR is shorten from 10.6 to 9.3 min, 67.9 to 63.3 min, respectively, compared with the traditional correction model. In addition, both the traditional and improved CMB model have a better performance in these aspects compared with the model which does not account for the CMB correction.

## 1. Introduction

The Chinese BeiDou navigation satellite system (abbreviated as BDS or BeiDou) is an important constituent part of the Global Navigation Satellite Systems (GNSS). Different from GPS, GLONASS and GALILEO, BDS satellites run not only in medium-altitude Earth orbit (MEO), but also in geostationary orbit (GEO) and inclined geosynchronous orbit (IGSO). BDS is the first satellite system which transmits triple-frequency signals on all satellites in operation. It has already launched a regional navigation service by the end of 2012. Now BDS is on its third step to develop a global system and provide global service in the near future. Presently, (at the beginning of 2018), a total of 14 BeiDou-2 satellites including five GEO, six IGSO, and three MEO satellites is in operation for public use, transmitting triple-frequency signals centered at B1 (1561.098 MHz), B2 (1207.14 MHz), and B3 (1268.52 MHz).

BDS has attracted the attention of many research groups and institutions in the fields of precise orbit and clock determination [1,2], triple-frequency ambiguity resolution (AR) in relative-positioning [3,4] or precise point positioning (PPP) [5,6,7], and so on. A large amount of research on these topics using the BDS observation has been performed. However, a recent study revealed that the systematic code-phase divergences, or code multipath bias (CMB), which are absent for GPS, GLONASS, Galileo and BeiDou-3, are commonly found in BeiDou-2 IGSO and MEO satellites [8,9,10,11,12]. It is found that the systematic bias is elevation-dependent, and varies by 0.4–0.6 m from horizon to zenith, being particularly pronounced for the B1 signal. Since such systematic bias was consistently observed with other receivers and antennas, it was, therefore attributed to the transmitting satellites. These systematic CMB variations are much larger than the code noise and would significantly enlarge the pseduorange errors. As a result, the performance of all precise data processing which uses code measurements, such as single-frequency and dual-frequency PPP, wide-lane (WL) ambiguity resolution based on Melbourne-Wübbena (MW) combination, would be severely affected.

Wanninger and Beer [9] identified two groups of BeiDou satellites (MEO and IGSO) whose signals are influenced in a similar way. These code biases were found to have no dependences on receiver type, time of observation, or satellite azimuth, but rather to be frequency- and elevation-dependent. Using the observation data of a set of globally distributed receivers, they determined the corrections and demonstrated that the application of these corrections successfully cures this deficiency. Guo et al. [13] used more datasets (covering a time span of almost two years) to produce the correction values together with the stochastic information, i.e., the precision indexes for the purpose of refining the stochastic model of code observations. They limited the elevation angles to 5–85° with a node separation of 10°. Validation tests in PPP reveal the actual precision of the corrected code observations can be reflected in a more objective manner if the stochastic model of the corrections is taken into account. Lou et al. [4] modeled the single-differenced CMB with widespread ground stations since the elevation of a GEO satellite remains unchanged. Their results suggest that the code bias variations have little impact on the resolution of extra wide-lane ambiguities due to its long wavelength, and also that of narrow-lane (NL) AR based on geometry-based model. The WL AR success rate can be improved about 28% after applying the code bias variations corrections based on the overall success rate statistics for GEO, IGSO and MEO satellites. Geng et al. [1] investigated the impacts of the CMB correction models presented by Wanninger and Beer on BDS double differenced AR. Due to the dependence of the CMB on elevation angle, the lower orbit satellites, i.e., MEO satellites, and longer baselines were largely affected by CMB on WL AR. The comparison suggests that the CMB is not negligible for BDS AR in precise orbit determination, can be applied to improve IGSO and MEO satellites orbital products. The CMB corrections provided by Wanninger and Beer have been used by Li et al. [7] to fix the BDS IGSO+MEO ambiguities in PPP. As reported, in the WL fractional cycle bias (FCB) estimation, there are a few satellites whose WL float ambiguities usage rates were obviously lower than the average usage rate for all BDS satellites. It is indicated the CMB correction models probably could be further improved by estimating independent parameters for each of the IGSO + MEO satellites.

Nevertheless, all models mentioned above estimated two sets of corrections for IGSO and MEO satellites, respectively (also for GEO in Lou et al. [4]). However, the WL FCB estimation with CMB indicated that the correction effectiveness slightly varies for different satellite. Hence it is suggested to estimate the corrections for each BDS IGSO and MEO satellite. In addition, as shown in the results of Wanninger and Beer, the corrections during elevation session between 0 and 10, and between 80 and 90° didn’t strictly obey a linear variation against the elevation. For this reason, a denser elevation node may be better to model the CMB variations. Currently, the institutions such as IGS-MGEX operating a global distributed network can provide the daily BDS observations from over 120 stations. These large amounts of data provide adequate support to refine the CMB estimation satellite by satellite with a denser elevation node separation.

In this study, the method proposed by Wanninger and Beer is further modified and extended to estimate the CMB corrections at 5° elevation node for each BDS IGSO + MEO satellite. One month of BDS data from all BDS stations from MGEX are used in this study to conduct the experiments. The effectiveness of our corrections is also assessed by means of WL FCB estimation and BDS PPP AR, compared with the results using the corrections by Wanninger and Beer and the results ignoring the CMB correction. This paper is organized as follows: the mathematical models for BDS code multipath bias estimation and the description of the improved method are given in Section 2. In Section 3, the effectiveness of the proposed model is validated by WL FCB estimation and dual-frequency BDS PPP AR. The conclusions are given in Section 4.

## 2. Methods

In this section, starting with the basic BDS observational equations, the multipath and MW linear combination are derived for the CMB estimation and analysis. Then a detailed description of our CMB estimation method is given.

### 2.1. Observation Equations and Linear Combination

The functional model describing code and carrier-phase observables can be expressed as [5,6]:

(1) $$P_{r,j}^{s}=\rho_{r}^{s}+c(dt_{r}^{}-dt^{s})+T^{s}+u_{j}I_{r}^{s}+B_{r,j}^{}-B_{j}^{s}+e_{r,j}^{s}$$

(2) $$\lambda_{j}^{}\phi_{r,j}^{s}=\rho_{r}^{s}+c(dt_{r}^{}-dt^{s})+T^{s}-u_{j}I_{r}^{s}+\lambda_{j}^{}(N_{r,j}^{s}+b_{r,j}^{}-b_{j}^{s})+\varepsilon_{r,j}^{s}$$

where *s* refers to one BDS satellite, *r* refers to the receiver, the subscript *j* refers to a given frequency; *P* is the code measurement (unit: m); *φ* is the carrier-phase measurement (unit: cycle); *λ* is the wavelength of carrier phase; *ρ* is the geometric distance between the phase centers of the satellite and receiver antennas, including displacements due to Earth tides and ocean loading and relativistic effects; *c* is the speed of light; *dt_r_* and *dt^s^* are the clock errors of receiver and satellite, respectively; *T^s^* is the slant troposphere delay; *u_j_* is a constant = $f_{1}^{2}/f_{j}^{2}$, where *f_j_* is the frequency of the *j* carrier; $I_{r}^{s}$ is the slant ionospheric delay at the first frequency; *N* is the integer ambiguity; *b_r_* and *b^s^* are the receiver-dependent and satellite-dependent fractional cycle bias, respectively; *B_r_* is the signal delay from receiver antenna to the signal correlator in the receiver; *B^s^* is signal delay from satellite signal generation to signal transmission from satellite antenna; *e* is the pseudorange measurement noise; *ε* is measurement noise of carrier phase.

With the dual- or triple-frequency code and carrier phase observations, the following multipath linear combination is popular used to analyze and assess the multipath effects on the code observation [9]:

(3) $$MP_{i}=p_{i}-\frac{f_{i}^{2}+f_{j}^{2}}{f_{i}^{2}-f_{j}^{2}}\lambda_{i}\phi_{i}+\frac{2f_{j}^{2}}{f_{i}^{2}-f_{j}^{2}}\lambda_{j}\phi_{j}$$

The linear coefficients are selected in such a way that ionospheric and tropospheric delay as well as all geometric contributions cancel out. Hence the multipath combination as shown in Equation (3) is an ionosphere-free and geometry-free combination. It mainly consists of code multipath, a constant ambiguity term which is a combination of the ambiguities of the two phase measurements, a combined signal delay, and observation noise. The theoretical maximum multipath effect on carrier phase observable is only one quarter of carrier wave cycle. For BDS satellites, based on Equation (3), the maximum carrier phase multipath effects on B1, B2 and B3 frequency multipath combination is not over 10 cm. Compared with the size of code multipath that is at the level of several meters, the carrier phase multipath effects are negligible [8]. Subtraction of the mean value from the measurements removes the phase ambiguities, which are constant if there are no cycle slips. Long-term changes in the range differences between the pseudoranges and carrier phase measurements can be detected in multipath variations. However, it is worth mentioning that the absolute values of multipath are unknown. Only variations of code multipath are available from the multipath series.

The multipath combination above serves for the BDS CMB analysis in this study. It should be noted that for the multipath combination at frequency *i*, a reference frequency *j* which is different from *i* need to be selected. Currently, the number of dual-frequency BDS receiver is much more than that of triple-frequency BDS receiver in the MGEX network. Hence, it is better to form the multipath combination with the B1 or B2 frequency as the reference one.

The frequently-used MW combination of code and carrier phase measurements [14,15], given as:

(4) $$MW=\left(\phi_{r,1}^{s}-\phi_{r,2}^{s}\right)-\left(f_{1}\cdot P_{r,1}^{s}+f_{2}\cdot P_{r,2}^{s}\right)\left(f_{1}-f_{2}\right)/\left(f_{1}+f_{2}\right)/c$$

is the other kind of linear combination which is heavily impacted by the code multipath. It cancels not only the non-dispersive effects, but also the ionospheric refraction. The MW combination provides a noisy estimation of the wide-lane ambiguity with a long wavelength of 84.7 cm for BDS (86.2 cm for GPS). However, the resulting signal is affected by the code multipath and pseudorange noise, which can reach up to several metres.

Duo to these properties, the MW combination is often used for cycle slip detection [16]. It can be used to detect the large cycle slips of over two cycles. It can also serve for fixing the wide-lane ambiguity using the smoothed value over a continuous arc. In PPP, the WL ambiguity fixing requires the FCB correction in order to remove the fractional parts of the single-differenced ambiguities between satellites [7]. Different from the multipath combination, the MW combination contains the linear combination of the CMB on B1 and B2 frequency. It is rank-defect to estimate the corrections for each frequency using the MW combination. Hence, the B1 and B2 CMB cannot be estimated with the MW combinations at the same time. Instead, the MW combination could be used for CMB analysis and assessment.

### 2.2. A New Improved Correction Model

The basic idea of CMB estimation is to best fit the systematic residual from the multipath combinations with the constant value subtracted. The constant value, theoretically a combination of the carrier phase ambiguity, however, is unknown. We use the averaged value of the arc instead. As discussed above in Section 1, most existed models estimated two sets of corrections for IGSO and MEO satellites, respectively (also for GEO in Lou et al. [4]). However, with the traditional CMB corrections in the WL FCB estimation, there are a few satellites whose WL float ambiguities usage rates were obviously lower than the average usage rate for all BDS satellites [7]. It indicated that the correction effectiveness slightly varies for different satellite. Hence it is suggested to estimate the corrections for each BDS satellite. In addition, different from most existed models above which performs estimation with an elevation node separation of 10°, we estimate the CMB with an elevation node separation of 5° in order to model the CMB variations within a small scale of elevation more precisely.

For this reason, the CMB estimation method proposed by Wanninger and Beer et al. [9] is modified and then applied to generate the new CMP corrections as follows: elevation dependent piecewise linear function with an elevation node separation of 5° were used to model the CMB; the corrections were estimated for each BDS IGSO + MEO satellite. In our proposed model, the number of parameters is about 10 times as much as that in the traditional model. Currently, the institutions such as IGS-MGEX operated over 120 stations which can provide the daily BDS observations. These large amounts of data can be well involved for the CMB estimation satellite by satellite. With all multipath combinations in each continuous arc tracked by a BDS station network, the equation sets in the form of (5) can be formed:

(5) $$MP_{r}^{s}=A_{r}^{s}+a_{m}\cdot x_{m}^{s}+\left(1-a_{m}\right)\cdot x_{m+1}^{s}$$

where A is the ‘true’ value of a multipath combination arc; $a_{m}$ is the coefficient corresponding to the CMB at the elevation node m; $x_{m}^{s}$ and $x_{m+1}^{s}$ denote the CMB parameter at the elevation node m and m+1, respectively.

**Figure 1:**
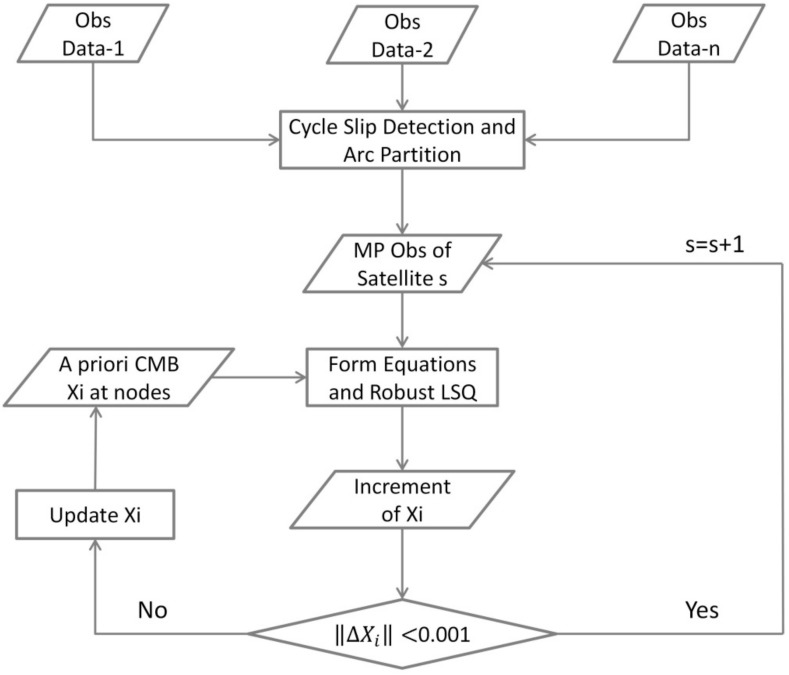
The flowchart of our CMB estimation procedure.

A flowchart of our CMB estimation procedure is shown in Figure 1 while the description of the model and parameter processing is given in Table 1. First, Turbo-edit algorithm using the MW linear combination, together with the carrier phase geometry-free combination, is applied to detect cycle-slips [16]. Second, the multipath combinations of each continuous arc are derived. The coefficients of the piecewise linear model are calculated according to the elevation angle. Because the value of A is unknown to us. Instead, we calculate *A* by averaging the multipath arc with the CMB corrected. Hence the iterative least squares algorithm is used to estimate the CMB increment corresponding to the CMB initial value and then get the precise result of *A*. During the iteration, only when the module of the CMB increment is less than 0.1 cm, the procedure ends and the final CMB values are derived. In order to eliminate the rank deficiency, we add a constraint that the sum of the corrections equals zero for each satellite at each frequency. In order to mitigate the impact of possible biases, caused by for example, large multipath effects, un-detected cycle slip, on the parameter estimation, the multipath observation with a large residual will be down-weighted during the iteration.

## 3. Experiment Analyses

We aim to assess the performance of our proposed CMB estimation method using three groups of experiments. Firstly, the traditional and the improved CMB corrections are directly compared to indicate the CMB variations between the satellites at the same orbit type. Then, we estimated the WL FCB without corrections, with the traditional and improved CMB estimations (referring to solution-A, -B and -C for the sake of brevity), respectively. The usage rate, namely the percentage of the valid WL ambiguities contributed to FCB estimation among all the input WL ambiguities, is compared. At last, the BDS PPP AR is conducted in solution A, B and C in static mode. The time to first fix (TTFF) [17,18] and the positioning bias are compared.

**Figure 2:**
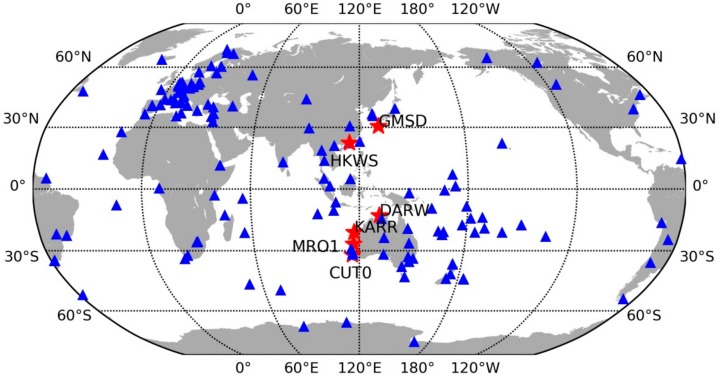
Distribution of the BDS reference network and user test stations. The blue triangles denote the reference stations used for FCB estimations; the red stars denote the user stations used for investigating the performance of the proposed CMB model.

The BDS observations, recorded at 30 s sampling intervals from IGS-MGEX [19], were used for our experimental analysis. Figure 2 shows the distribution of the BDS reference network and user stations. Approximately 150 stations were used for CMB and FCB estimation. Six stations denoted by red stars were used for the experimental tests. Site information for six user stations, including the site name, receiver type, antenna type, were summarized in Table 2. The daily observations from DOY 335 to 365, 2017, were used in this study. At the user end, daily observables were separated into 12 2-h-long observable sessions for experiments. The 2-h-long observable was removed if its average visible BDS IGSO + MEO satellite is less than 5. In total there were about 2000 tests finally used for experiments.

For dual-frequency FCB estimation, the WL FCB is estimated on a daily basis while the narrow-lane (NL) FCB every 15-min, as suggested by [7]. Due to its orbit property, it is reported that the orbit and clock error for BDS GEO is much larger than that of BDS IGSO + MEO, and the elevation variations of GEO satellites are very small. Therefore, we didn’t estimate the CMB for BDS GEO satellites in this study. Only the CMB and FCB for the BDS IGSO + MEO satellites are estimated at server end and the ambiguity of BDS IGSO+MEO is aimed to be fixed in the user end. With the estimated single-differenced FCBs, the integer property of single-differenced BDS IGSO + MEO ambiguities can be recovered. We perform single differencing between satellites operation on ambiguity level in practice as it provides more flexibility of the choice of a reference satellite and the formation of satellite pairs. The WL float ambiguities can be directly fixed by the rounding approach due to its long wavelength [20]. For NL float ambiguities, they are fed into the Least-squares AMBiguity Decorrelation Adjustment (LAMBDA) method algorithm to search for the best integer solutions because there exists strong correlation between the PPP narrow-lane ambiguities [21]. Partial ambiguity resolution strategy is employed to increase the probability of a successful fixing [18,22]. A fix solution can be obtained at user end once both of the WL and NL integer ambiguities are fixed. For ambiguity validation, the ratio-test given by the following expression is used [23,24,25]:

(6) $$ratio=\frac{{\left\|N-n_{2}\right\|}_{Q_{N}}^{2}}{{\left\|N-n_{1}\right\|}_{Q_{N}}^{2}}$$

where ${\left\|\cdot \right\|}^{2}$ denotes the calculation of squared norm; N is the float PPP ambiguity with the variance-covariance matrix $Q_{N}$; $n_{1}$ and $n_{2}$ is the best and second-best ambiguity integer candidate, respectively.

The ratio test in fact tests the closeness of the float solution to its nearest integer vector. A larger ratio value indicates a more reliable ambiguity resolution [23]. In this study, the threshold is set to 3 as suggested by Leick [24]. In theory, the ratio test with a fixed failure rate is more suitable for the GNSS ambiguity resolution [25]. However, the fixed failure rate approach uses Monte Carlo sampling to create a look-up table. It is time-consuming due to the characteristics of Monte Carlo sampling and in order to make the table be valid for a general scenario. A detailed description of the observation model, error processing and estimated parameters for BDS PPP is given in Table 3. 

The combined precise GPS and BDS satellite orbit and clock products provided by GFZ (with a prefix ‘GBM’) were used for PPP processing [26]. To maintain consistency with the GBM precise products, the satellite phase center offset (PCO) and variation (PCV) corrections estimated by ESA were applied for BDS. For receiver PCO and PCV corrections, there were no values provided for BDS signals at this time, we simply used GPS corrections for BDS signals. This processing strategy is consistent with the strategy employed for BDS precise orbit determination and clock estimation [4]. The elevation-dependent weighting scheme of observations applied in this research to mitigate the effects of multipath as well as atmospheric errors is given as [7]:

(7) $$\sigma^{2}=a^{2}+a^{2}/\sin^{2}\left(el\right)$$

where σ is the standard deviation of the BDS measurements and el is the elevation angle of the satellite. For carrier phase observations, the values of a for the BDS IGSO + MEO and BDS GEO were set to 3 and 10 mm, respectively while for code observations, the values of a for the BDS IGSO + MEO and BDS GEO were set to 0.3 m, and 1.0 m, respectively.

### 3.1. Evaluation of CMP Estimation

**Figure 3:**
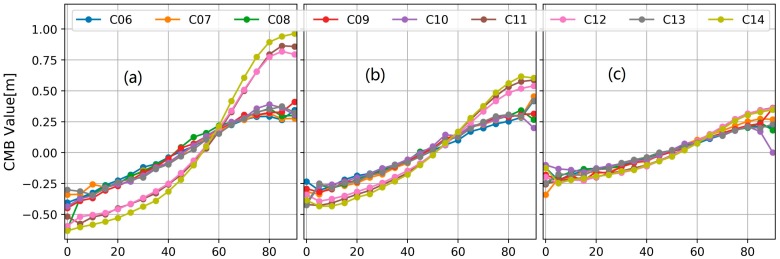
The CMB values estimated by our proposed model. (**a**) the values at the B1 frequency; (**b**) the values at the B2 frequency; (**c**) the values at the B3 frequency.

Firstly, our CMB corrections at every 5° elevation node for B1, B2 and B3 are shown in Figure 3. As we can see, the code measurements of MEO satellites tracked on B1 frequency at high elevations are seriously affected by the code bias, which reaches close to 1.0 m especially when the elevation rises up to 80°. The IGSO satellites are less impacted by the CMBs than the MEO satellites especially at B3 and B2 frequency. Besides, for each BDS IGSO + MEO satellite, the code biases on B2 are much smaller than those on B1 and those on B3 frequency are smallest. More importantly, it is found that the B1 CMB of C14 is obviously different from that of other MEO satellite C11 and C12. For the elevation range 0–30°, the B1 CMB of C14 is smaller than other MEO satellites while for the range 70–90° its value is larger than those with a difference close to 0.1 m. These results demonstrated the existence of the CMB difference even for the satellites at the same orbit type. It indicated that it is more reasonable to estimate the CMB corrections for each BDS IGSO + MEO satellite.

**Figure 4:**
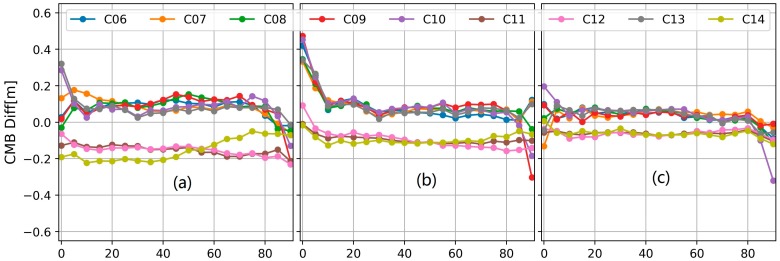
The differences of the CMB values between our proposed model and the traditional model. (**a**) the differences at the B1 frequency; (**b**) the differences at the B2 frequency; (**c**) the differences at the B3 frequency.

Furthermore, the corrections given by Lambert and Beer are interpolated to get the values at every 5° elevation node. Then, the differences between the traditional and improved correction are calculated and shown in Figure 4. Caused by the different parameter constraint used in the traditional and our improved method, the CMB difference may be systematic and not close zero. The inconsistency of the two models is mainly reflected by the variational part of the CMB difference.

As shown in Figure 4, a significant model inconsistency was observed for the elevation below 5° and over 85°. This is because for CMB estimation, the multipath combination with low elevation is heavily down-weighed and the number of multipath combination with high elevation is relatively less. For the elevation between 10 to 80°, the B3 CMBs from two models have a good consistency with each other. However, for the B1 frequency, the CMB difference of C14 increased from about −0.2 m to about −0.05 m while for C11 and C12, the B1 CMB difference decreased from about −0.1 m to −0.2 m. Also the B1 CMB difference variations of the IGSO satellites are slightly different from each other.

### 3.2. WL FCB Quality

**Figure 5:**
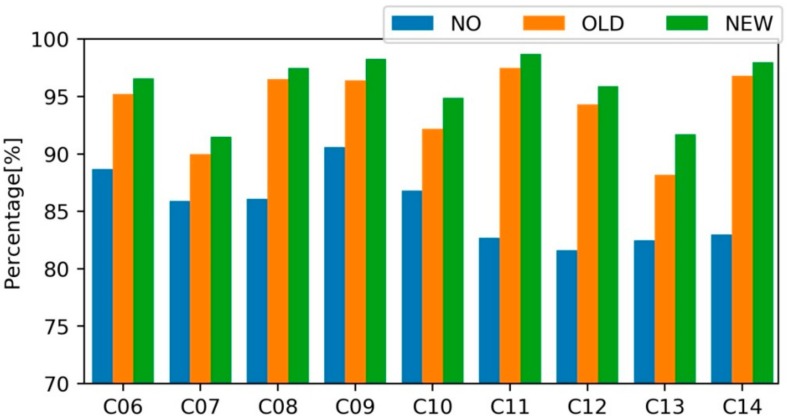
Usage rate of the BDS WL float ambiguities without (NO), with the traditional (OLD) and the new CMB corrections, respectively.

As discussed above, the MW combination could be affected by the code multipath biases. As a result, the WL FCB which is estimated using the MW combination would be affected. We estimated the daily WL FCB for solution A, B and C, respectively, and then calculate the average usage rate for each BDS IGSO + MEO satellite. The higher usage rate, the better the FCB consistency is. Figure 5 shows the average usage rate of WL ambiguities for each satellite.

As shown in Figure 5, the WL usage rate of the solution A is generally lower than 90% except for C09 whose rate is 90.6%. C12 has a lowest usage ratio of 81.6% among all nine BDS IGSO + MEO satellites. With the traditional model, the usage rate of solution-B ranges from 88.2% to 97.5% for an individual satellite and is significantly improved compared with solution A. The improved model further increases the usage rate of each satellite. For solution-C, the WL usage rate of BDS is between 91.5% and 98.7%. The average usage rate value for all BDS satellites is 85.3, 94.1 and 96.0% for solution-A, B and C, respectively. By comparing the usage rates of BDS WL ambiguities for FCB estimation, it was indicated that the satellite-induced CMB has a significant influence on the WL ambiguity calculation and our new model works well to mitigate the influence of the CMB on MW-based WL ambiguity calculation. It outperforms over the traditional model which considering the bias only orbit-type related while satellite-independent, and with a node separation of 10°.

### 3.3. PPP Test

**Figure 6:**
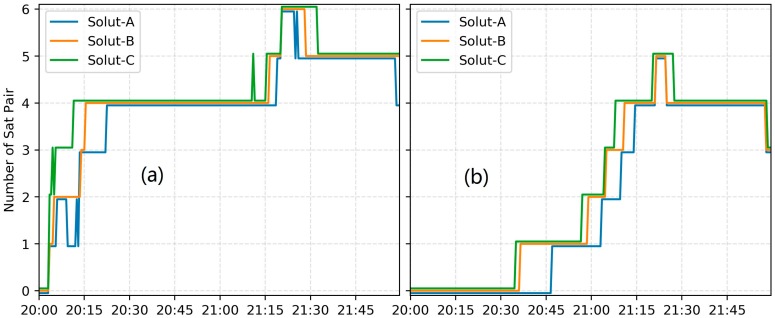
Time series of the number of BDS WL and NL resolved ambiguities for solution A, B and C, respectively. (**a**) the number of BDS WL resolved ambiguities; (**b**) the number of BDS NL resolved ambiguities.

A crucial performance indicator of PPP AR is the TTFF. Figure 6 shows a typical BDS PPP AR TTFF results for solution A, B and C, respectively, taking the results for station CUT0, on DOY 352, 2017. In this study, only when the number of ambiguity-fixed single-differenced satellite pair is not less than 4, the fixed ambiguities would be accepted at this epoch. It takes 22.5, 15.0 and 11.0 min for the WL TTFF, while 74.5, 71 and 65.5 for the NL TTFF, in solution A, B and C, respectively. The comparison of solution A with B/C demonstrated that the TTFF of BDS PPP AR can be heavily impacted by CMB. Furthermore, it is found that our improved model can provide a better correction effect than the traditional one based on the comparison of solution B and C. We also calculated the GPS dual-frequency PPP AR solution for this observation with the GPS FCB estimated using the same reference network. The corresponding WL and NL TTFF is 6.0 and 23.5 min. It is reasonable that the GPS PPP AR has a faster TTFF mainly because the number of average visible GPS satellite is more than that of BDS IGSO + MEO [7].

Table 4 presents the statistical TTFF of the PPP AR tests. The WL average statistics is based on all the 2 h-session observations. Due to its long wavelength of about 84.7 cm, the WL ambiguity which is obtained by averaging over the MW combinations in a continuous arc is much easier to be fixed compared with the NL ambiguity. The WL ambiguities are mainly impacted by the code noise and the CMB. For solution A, the WL TTFF is generally 10–19 min. It is obviously longer than that of solution B and C because it takes a certain period to mitigate the impact of CMB. For solution B, approximately 7–16 min is required to achieve the first fixed solution. The solution C achieves the fastest TTFF among all three groups of solutions, approximately 5–14 min. The averaged WL TTFF is 13.8, 10.6 and 9.3 min for solution A/B/C respectively. The improved model shortens the WL TTFF by about 32.6% compared with the results solution A while 12.3% compared with that of solution B.

Different from the WL results, the NL average statistics above is only based on the 2h-session file which can be fixed. It’s worth noting that the number of session files which can achieve an ambiguity-fixed result for solution A, B and C is 1330, 1560 and 1620, respectively. Therefore, compared with the traditional model, the improved model can increase the possibility of a 2h-session observation to be fixed. Moreover, the NL TTFF can also be accelerated with the improved CMB model. The averaged NL TTFF is 73.5, 67.9 and 63.3 min for solution A/B/C respectively. The improved model shortens the NL TTFF by about 13.9% compared with the results solution A while 6.8% compared with that of solution B. The average BDS NL TTFF is much longer than the GPS NL TTFF which is generally about 30 min, mainly because that the number of visible GPS satellite is sufficient and more than that of BDS IGSO + MEO. In the near future, with the global deployment of BDS, the NL TTFF of BDS PPP AR is expected to be much improved.

Using the 2 h static PPP positioning bias results over all test days, the bias RMS in east, north and up direction is calculated for each station and is given in Table 5. One can see that currently BDS static PPP with 2 h observation can achieve an accuracy of several centimetres in three components and the smallest RMS is in the north direction and the largest RMS is in the up direction. The RMS of all 2 h solutions was significantly improved with CMB corrected. With our improved model, the highest positioning accuracy was achieved by solution C which is 3.8, 2.6, 5.8 centimetres in the east, north and up directions, respectively. The improvement of the positioning accuracy brought by our proposed method is 15.6, 21.2 and 13.4% compared with solution A, while 7.3, 7.1 and 6.5% compared with solution B, in the east, north and up directions, respectively.

## 4. Conclusions

Different from the other GNSS systems, BeiDou-2 (IGSO and MEO) code measurements are polluted by the satellite-induced systematic code multipath bias. In this study, an improved method which estimates the bias for each IGSO + MEO satellite with an elevation node session of 5° is proposed in this study. The continuous piecewise linear functions were employed to produce the correction values. One month of BDS observations recorded on a globally distributed MGEX stations equipped with different receiver types were used.

Three groups of tests, namely, evaluation of CMB estimation, the ambiguity usage for WL FCB estimation, and the performance of PPP AR (TTFF and positioning bias), were conducted to verify the effectiveness of the proposed model and compare the performance of CMB correction with the traditional model. Experimental results show that for the satellites on the same orbit type, obvious difference can be found in the CMB at the same node and frequency. The new correction model outperforms the old one assuming that for each frequency, the same CMB corrections are suitable for the BeiDou-2 satellites operating in the same orbit type. The new correction model can improve the WL ambiguity usage rate for WL FCB estimation, shorten the WL and NL TTFF in PPP AR as well as improve the PPP positioning accuracy. With the new correction model, the usage of WL ambiguity for FCB estimation is increased from 94.1% to 96.0%, the WL and NL TTFF of PPP AR is shorten from 10.6 to 9.3 min, 67.9 to 63.3 min, respectively, compared with the traditional correction model. The positioning accuracy, as well as TTFF, was obviously enhanced by the improved model. In addition, both the traditional and improved CMB model have a better performance in these aspects compared with the model which not accounting for the CMB correction.

## Figures and Tables

**Table 1:** Models and parameters for BDS CMB estimation.

Items	Models
Observation	Multipath combination
Sampling rate	30 s
Estimator	Iterative Least Square with robust estimation
Modelling	Satellite-independent, elevation-dependent;
	Piecewise linear
Weighting scheme	Elevation-dependent (Equation (7))
Cycle-slip detection	Turbo-edit
Parameter estimation	One parameter at each 5° node

**Table 2:** Site information of six user stations. The information includes the name, receiver type, antenna type.

Station	Receiver Type	Antenna Type
CUT0	TRIMBLE NETR9	TRM59,800.00 SCIS
DARW	SEPT POLARX5	JAVRINGANT_DM NONE
GMSD	TRIMBLE NETR9	TRM59,800.00 SCIS
HKWS	LEICA GR50	LEIAR25.R4 LEIT
KARR	TRIMBLE NETR9	TRM59,800.00 NONE
MRO1	TRIMBLE NETR9	TRM59,800.00 NONE

**Table 3:** Observation models, error processing, and estimated parameters for BDS PPP.

Items	Models
Observation	B1/B2 ionosphere-free code and carrier phase
Sampling rate	30 s
Elevation cutoff	10°
Weighting scheme	Elevation-dependent (Equation (7))
Error correction	Zenith troposphere dry component;
	Relativistic effect;
	Solid Earth tide;
	Satellite and receiver PCO and PCV;
	Phase-windup;
	Satellite FCB (corrected for ambiguity-fixed PPP)
Parameter estimation	Coordinate (epoch-wise);
	Receiver clock (epoch-wise)
	ZWD (random-walk, with a)
	Ambiguities (Constant for each arc)

**Table 4:** The average TTFF (min) for kinematic PPP AR.

SITE	WL TTFF			NL TTFF		
	Solution A	Solution B	Solution C	Solution A	Solution B	Solution C
CUT0	18.8	16.0	14.2	79.3	69.1	62.2
DARW	10.3	7.8	7.1	71.1	68.8	62.7
GMSD	17.8	14.1	13.0	71.7	68.3	65.0
HKWS	14.7	11.2	9.8	69.8	66.0	63.4
KARR	9.8	6.7	5.2	72.8	67.5	64.1
MRO1	11.0	7.8	6.7	76.4	67.4	63.2
Average	13.8	10.6	9.3	73.5	67.9	63.3

**Table 5:** The averaged positioning bias RMS with 2 h observation for each test stations.

SITE	Solution A			Solution B			Solution C		
	E	N	U	E	N	U	E	N	U
CUT0	4.2	3.4	5.9	4.0	3.1	5.4	3.7	2.7	5.0
DARW	7.4	4.8	8.2	6.3	3.8	7.1	5.7	3.3	6.3
GMSD	3.5	2.6	6.7	3.3	2.4	6.2	3.3	2.2	5.9
HKWS	4.1	2.9	5.3	3.8	2.7	5.1	3.6	2.5	4.9
KARR	3.9	3.0	6.8	3.5	2.7	6.5	3.3	2.4	6.2
MRO1	3.8	2.7	7.1	3.5	2.5	6.9	3.3	2.4	6.5
Average	4.5	3.3	6.7	4.1	2.8	6.2	3.8	2.6	5.8
